# Selections

**Published:** 1860-05

**Authors:** 


### Selections.

---

Sanitary Science.—What Sanitary Science has done, and has yet to do, may be gathered from the following facts : The science is quite of modern date ; but since the application of its simplest principles, the cleansing of streams, drainage of houses, and introduction of pure water, the following evidence of benefits resulting has been given us : In Liverpool the mortality had fallen from 37 in the 1000 to 27 ; in Bradford from 28½ to 22 ; in Gloucester, from 27 to 24. Taking an average of nineteen towns which had been treated in this way, it was found that the death-rate dropped from 28 in the 1000 to 21. Corydon was taken in hand scientifically some time ago ; and since then an average of 196 lives have been saved in the town every year ! The mortality among the pauper infants and pauper children in the metropolitan unions has been enormously reduced. In the Military School at Chelsea a death-rate of nine in the thousand has been brought down to one of four. The female prisoners at Brixton, who live under sanitary rules, are three times as healthy as the poor needle-women of London ; and at Pentonville, notwithstanding the allowance to be made for moral depression, the death rate is only one-third of that prevailing in populous towns. But still there is a great work to be done ; for as we are told, authoritatively, that at least 100,000 persons die annually in these islands at premature periods and by preventible deaths ; and at least 1,000,000 more are wasted and debilitated from similar causes. Talk of war, indeed ! why, what battle or contests ever wrought havoc like this; havoc, be it remembered, not occurring at intervals, like an exceptioned calamity, but carried steadily and incessantly through the ranks of our population? And we have still to remember the lesson taught us by the Crimean war. In that war we lost altogether 20,800 men; but of this number 5,000 only were slain by the enemy. All the rest, 15,800 soldiers, fell victims to privation and disease.—*Medical Times and Gazette.*

---

Dr. Evans, the eminent American dentist residing in Paris, is thus spoken of by the Paris correspondent of the *Evening Post:*

"There are not many of our readers who have not heard of Dr. Evans, the American dentist, as he is called, *par excellence*, and who has charge of the teeth of pretty much all the sovereigns of Europe, and of America too, I suppose, when the latter come to Paris. He made a flying professional visit the other day to Nice, at the instance of the former Empress of Russia. On parting, she presented him with a diamond ring, valued at 60,000 francs, about $12,000. The Doctor and Mrs. Evans have received presents enough from crowned heads, in the shape of bracelets, watches, snuff-boxes, rings, and curious articles in gold and precious stones, to make him a millionaire, if he had their cost in money. There is, as I have already said, scarcely a sovereign on the continent of Europe from whom he has not some costly testimonial."

---

Proportion of the Sexes in Providence.—Dr. Snow, City Registrar of Providence, R. I., says: Of the 1,593 children born in Providence in 1859, there were 825 males and 768 females. This gives 107 males to every 100 females, or 51.8 males and 48.2 females in each 100 children born. These are almost exactly the average proportions in this city during six years past.

But there is a most remarkable difference in this respect between the American and foreign children, the explanation of which is not so obvious. Of the children of American parents, born in 1859, there were 102 males to 100 females, while of the children of foreign parents there were 111 males to 100 females. Can those who are interested in the subject give any explanation of this remarkable difference?—*Medical and Surgical Reporter.*

Administration of Anæsthetics during Sleep.—To avoid excitement and alarm in children, it may be very desirable to produce anæsthesia during sleep. To accomplish this without waking a sleeping patient, a very gradual and cautious administration is necessary. Dr. J. H. Beech states, in the *Peninsular and Independent Medical Journal*, that he has thus succeeded several times. One of these cases was the extraction of a grain of corn from the nostril of a restive child, aged three years. Some ineffectual efforts had been previously made to remove it. A sponge moistened with chloroform was held near to the mouth, being careful not to touch the face. After the removal of the foreign body, the child, without being awakened, was allowed to continue its usual sleep until morning, when he awoke at the usual hour, unconscious of anything unusual having occurred.

Diseased Tusk of an Elephant.—Dr. J. C. White showed a section made near the base of a tusk of a large elephant, illustrating the effect of the passage of a rifle ball through the dentinal pulp, from side to side. The ball, which was a large one of wrought iron, probably entered the thin socket formed by the prolongation of the premaxillary bone in which the tooth was inserted, breaking through the tender pulpy cone, and the tooth wall of the other side, and spent its last force against the interior surface of the socket on the opposite side. It then fell back within the rollow it had formed, and became imbedded within the new growth it excited, which consisted of large, irregular-shaped layers and masses of osteo-dentine, a tissue which was secreted instead of ivory, probably in consequence of the irritation produced by the foreign body.—*Boston Medical aud Surgical Journal.*

Sulphate of Copper with Opium in Diarrhea from Teething.—Among other therapeutical news, we notice, in a recent number of *Schmidt's Jahrbücher*, the following formula, which Prof. Eisenmann, of Würzburg, has found very efficient in diarrhea of children from teething, viz.: Cupri sulphat. $\frac{1}{4}$ gr.; pulv. opii. $\frac{3}{4}$ gr.; pulv. sacch. q. s. Three times daily one such powder.

AN EASILY APPLIED TREATMENT OF SHORT-SIGHTEDNESS. —When FOLTZ, Professor of Physiology at Lyons, first published his article on the mechanical treatment of myopy in the *Gazette Medicale*, a year ago, we doubted the efficiency of his method, and did not deem it entitled to a place in the *Reporter's Periscope.* While we make it truly a "view around," over the whole medical world, it is our constant endeavor to make our Periscope reliable, as well as comprehensive, and drawing from the medical journals of every country, we are especially careful to exclude those reports of extraordinary and "miraculous" cases, cures and inventions, those "hoaxes" which to our regret, we have afterward seen going the round of our own journals as "foreign *intelligence*" (!?) But while thus carefully selecting from the vast garners which the foreign journals present to us, we never lose sight of anything that might really prove of benefit to our profession; and though withholding the empty stalks, we as *Reporter* simply, leave to our readers the final trial and judgment of the value of the grain.

The following method we can now publish, however, with the recommendation resulting from entire or partial success in three or four cases, that have come under our observation.

Short-sighted persons, in looking at distant objects, usually blink, i. e. bring the upper and under eyelids nearer together, covering a part of the cornea. A finger is applied to the outer commissure of the lids in this position, and they are drawn outward a little. By this procedure the cornea is flattened, and the axis of the eye shortened. In consequence, the object looked at will appear surprisingly distinct in its outlines, often as much so, as if seen through suitable concave glasses. The pressure thus exerted through the lids on the eye must only be slight; when too great, the eye becomes presbyoptic, and vision dulled. Practice soon insures the exercise of the proper degree of pressure.

This simple, not painful, and entirely harmless proceeding is to the short-sighted of the same service as the use of the monocle or single eye-glass. It enables him to see distant objects sharply defined, to read signs, names of streets, numbers of houses, to recognize actors on the stage, or persons in a large hall, etc. The vast advantage of this "easy method" over concave glasses consists (besides its being always at hand), in its having not only palliative, but also curative effects. It has certainly lessened short-sightedness in our own case. The cornea partially regains through the slight but oft-repeated pressure on it, the natural retractility, lost through strong pressure from within ; its convexity no longer increases, but rather diminishes, as also the antero-posterior axis of the eye. It is not to be overlooked, how very easy of execution this gymnastic eye-exercise is. It subjects the individual to so little inconvenience and interruption of occupation, that it is soon performed from habit, and almost unconsciously.—*Medical and Surgical Reporter.*

